# Supplementary material for: Impact of Ligand Substitution and Metal Node Exchange in the Electronic Properties of Scandium Terephthalate Frameworks
Source: Inorg Chem. 2024 Jan 17;63(4):2098–108. doi: 10.1021/acs.inorgchem.3c03945 (PMC10828989; doi:10.1021/acs.inorgchem.3c03945)
Supplement: Supplementary file 1 — ic3c03945_si_001.pdf [file ic3c03945_si_001.pdf]

SUPPORTING INFORMATION

Impact of Ligand Substitution and Metal Node Exchange in the Electronic Properties of Scandium Terephthalate Frameworks

Holger-Dietrich Saßnick,[†] Fabiana Machado Ferreira De Araujo,[†,¶] Joshua Edzards,[†] and Caterina Cocchi[*,†,‡]

†*Institute of Physics, Carl-von-Ossietzy Universität Oldenburg, 26129 Oldenburg, Germany*

‡*Center for Nanoscale Dynamics (CeNaD), Carl-von-Ossietzy Universität Oldenburg, 26129 Oldenburg, Germany*

¶*Present Address: Institute of Materials Science, Technische Universität Darmstadt, 64289 Darmstadt, Germany*

E-mail: caterina.cocchi@uni-oldenburg.de

Structural Properties

Table S1: Bond lengths in Å between the metal atoms (Al, Sc, and Y) and the neighboring oxygen in the functionalized linker molecules at the $L1$ and $L2$ sites.

	Al		Sc		Y	
	$L1$	$L2$	$L1$	$L2$	$L1$	$L2$
H	1.892	1.892	2.100	2.094	2.252	2.251
CH_3	1.924	1.890	2.111	2.091	2.257	2.247
NO_2	1.912	1.895	2.107	2.093	2.261	2.253
Cl	1.914	1.890	2.109	2.089	2.259	2.246
Br	1.933	1.901	2.121	2.096	2.266	2.251
NH_2	1.904	1.883	2.100	2.088	2.240	2.251
OH	1.896	1.889	2.095	2.098	2.249	2.258
COOH	2.179	2.123	2.134	2.115	2.276	2.268

Table S2: O-C1-C1-O dihedral angles (in degrees) in the functionalized linker molecule at the $L1$ and $L2$ sites.

	Al		Sc		Y	
	$L1$	$L2$	$L1$	$L2$	$L1$	$L2$
H	40.46	0.74	40.46	0.85	37.21	0.88
CH_3	33.91	2.60	38.61	1.17	37.21	2.75
Cl	31.09	2.11	31.02	1.56	29.70	1.41
Br	27.94	2.02	28.05	1.76	25.36	1.56
COOH	53.83	23.58	30.12	3.60	35.00	2.61
OH	33.87	0.87	29.15	0.65	20.52	0.48
NO_2	26.96	1.91	29.63	2.78	27.33	6.67
NH_2	35.52	5.28	34.19	3.23	27.69	1.94

Table S3: Volumes of the relaxed unit cells of the considered MOFs with Al, Sc, and Y metal nodes with varying ligand functionalization and relative change in the volume (RC_V) in comparison to the H-terminated structures.

	Al		Sc		Y	
	Vol. / Å^3	RC_V / %	Vol. / Å^3	RC_V / %	Vol. / Å^3	RC_V / %
H	1416.17	0.00	1601.40	0.00	1747.32	0.00
CH_3	1448.96	2.32	1619.72	1.14	1752.15	0.28
Cl	1455.12	2.75	1626.39	1.56	1765.31	1.03
Br	1482.54	4.69	1644.30	2.68	1773.62	1.51
COOH	1521.02	7.40	1642.43	2.56	1773.19	1.48
OH	1424.07	0.56	1599.65	-0.11	1728.66	-1.07
NO_2	1447.47	2.21	1606.18	0.30	1739.31	-0.46
NH_2	1424.33	0.58	1598.79	-0.16	1740.80	-0.37

Table S4: Lengths in Å of the hydrogen bonds formed between the H atoms of the indicated functional groups and the O atoms in the BDC linker molecule at sites $L1$ and $L2$.

	Al		Sc		Y	
	$L1$	$L2$	$L1$	$L2$	$L1$	$L2$
COOH	–	–	–	1.436	–	1.455
OH	1.741	1.778	1.742	1.753	1.720	1.743
NH_2	2.019	1.962	2.014	1.952	2.015	1.962

Stability

Table S5: Elemental phases of the indicated atomic species used to calculate the formation energy of the considered MOFs taken from the Materials Project database.

	Space group	Materials Project ID	E_{tot}/atom (Ha)
Al	$Fm\bar{3}m$ [225]	mp-134	-2.079
Sc	$P6_3/mmc$ [194]	mp-67	-46.683
Y	$P6_3/mmc$ [194]	mp-112	-38.324
C	$P6_3/mmc$ [194]	mp-48	-5.700
O	$P4_12_12$ [92]	mp-723285	-127.658
H	$P6_3/mmc$ [194]	mp-24504	-0.584
N	$Pa\bar{3}$ [205]	mp-25	-9.955
Cl	$Cmca$ [64]	mp-22848	-14.975
Br	$Immm$ [71]	mp-998864	-13.421

Table S6: Formation energy (E_{form}) in eV/atom and energy gap (E_{gap}) in eV computed for all the considered MOFs.

	Al		Sc		Y	
	E_{form}	E_{gap}	E_{form}	E_{gap}	E_{form}	E_{gap}
H	-0.673	3.36	-0.741	3.04	-0.762	3.24
CH_3	-0.541	2.83	-0.597	2.60	-0.613	2.82
Cl	-0.659	2.26	-0.732	2.13	-0.754	2.40
Br	-0.597	1.69	-0.679	1.69	-0.705	2.04
COOH	-0.833	2.27	-0.901	2.21	-0.923	2.39
OH	-0.831	1.73	-0.893	1.35	-0.913	1.54
NO_2	-0.600	2.34	-0.660	2.31	-0.677	2.43
NH_2	-0.594	1.45	-0.649	1.15	-0.663	1.18

Partial Charge Analysis

Table S7: Partial atomic charges in e calculated via the Bader scheme and averaged over equivalent atomic sites for the Al-based MOFs with varying linger functionalization. The values obtained for the atoms in the ligand on sites $L1$ and $L2$ are reported separately. X stands for the functional group reported in the header.

		H	CH_3	Cl	Br	COOH	OH	NO_2	NH_2
Al		2.515	2.519	2.520	2.523	2.506	2.516	2.520	2.516
O	$L1$	-1.189	-1.187	-1.180	-1.180	-1.167	-1.194	-1.174	-1.195
	$L2$	-1.190	-1.192	-1.184	-1.185	-1.176	-1.197	-1.177	-1.200
C1	$L1$	1.413	1.396	1.438	1.436	1.429	1.369	1.447	1.374
	$L2$	1.441	1.431	1.468	1.462	1.441	1.401	1.487	1.401
C2	$L1$	-0.010	-0.016	0.019	0.017	0.022	0.009	0.053	-0.013
	$L2$	-0.012	-0.021	0.013	0.009	0.024	-0.000	0.047	-0.008
C3	$L1$	-0.004	0.005	0.090	-0.061	0.026	0.507	0.233	0.357
	$L2$	0.000	0.007	0.086	-0.076	0.023	0.531	0.245	0.376
C4	$L1$	0.025	0.018	0.060	0.058	0.051	0.046	0.090	0.017
	$L2$	-0.010	-0.023	0.009	0.006	0.016	0.000	0.042	-0.020
H	$L1$	0.031	0.020	0.053	0.047	0.057	0.046	0.078	0.022
	$L2$	0.067	0.066	0.104	0.105	0.088	0.077	0.099	0.063
X	$L1$	0.068	0.089	-0.159	0.004	-0.101	-0.446	-0.405	-0.228
	$L2$	0.063	0.095	-0.142	0.031	-0.066	-0.443	-0.400	-0.239

Table S8: Partial atomic charges in e calculated via the Bader scheme and averaged over equivalent atomic sites for the Sc-based MOFs with varying linger functionalization. The values obtained for the atoms in the ligand on sites $L1$ and $L2$ are reported separately. X stands for the functional group reported in the header.

		H	CH_3	Cl	Br	COOH	OH	NO_2	NH_2
Sc		2.120	2.119	2.123	2.126	2.128	2.113	2.126	2.110
O	$L1$	-1.116	-1.117	-1.110	-1.111	-1.105	-1.120	-1.101	-1.124
	$L2$	-1.119	-1.119	-1.112	-1.113	-1.110	-1.123	-1.107	-1.126
C1	$L1$	1.401	1.388	1.429	1.429	1.417	1.358	1.440	1.363
	$L2$	1.429	1.417	1.454	1.451	1.439	1.386	1.474	1.384
C2	$L1$	-0.008	-0.016	0.021	0.016	0.023	0.010	0.046	-0.012
	$L2$	-0.011	-0.018	0.013	0.010	0.023	-0.002	0.045	-0.008
C3	$L1$	0.002	0.005	0.084	-0.060	0.021	0.510	0.239	0.360
	$L2$	0.001	0.006	0.086	-0.073	0.023	0.531	0.240	0.380
C4	$L1$	0.022	0.021	0.065	0.060	0.053	0.046	0.083	0.016
	$L2$	-0.014	-0.023	0.009	0.008	0.018	-0.002	0.043	-0.020
H	$L1$	0.029	0.020	0.053	0.048	0.042	0.043	0.077	0.023
	$L2$	0.071	0.065	0.107	0.107	0.080	0.081	0.097	0.063
X	$L1$	0.065	0.090	-0.158	0.002	-0.065	-0.446	-0.399	-0.228
	$L2$	0.063	0.095	-0.144	0.023	-0.066	-0.445	-0.391	-0.239

Table S9: Partial atomic charges in e calculated via the Bader scheme and averaged over equivalent atomic sites for the Y-based MOFs with varying linger functionalization. The values obtained for the atoms in the ligand on sites $L1$ and $L2$ are reported separately. X stands for the functional group reported in the header.

		H	CH_3	Cl	Br	COOH	OH	NO_2	NH_2
Y		2.284	2.281	2.287	2.289	2.293	2.280	2.292	2.273
O	$L1$	-1.139	-1.140	-1.132	-1.134	-1.130	-1.142	-1.124	-1.147
	$L2$	-1.144	-1.144	-1.137	-1.138	-1.136	-1.145	-1.131	-1.150
C1	$L1$	1.402	1.389	1.430	1.429	1.420	1.356	1.442	1.362
	$L2$	1.427	1.416	1.453	1.451	1.437	1.382	1.467	1.381
C2	$L1$	-0.008	-0.017	0.019	0.018	0.021	0.011	0.046	-0.007
	$L2$	-0.011	-0.018	0.013	0.010	0.022	-0.004	0.044	-0.010
C3	$L1$	-0.003	0.010	0.087	-0.063	0.024	0.504	0.230	0.365
	$L2$	0.003	0.007	0.086	-0.070	0.020	0.525	0.237	0.379
C4	$L1$	0.024	0.017	0.063	0.060	0.053	0.047	0.084	0.020
	$L2$	-0.016	-0.026	0.007	0.007	0.019	-0.002	0.038	-0.028
H	$L1$	0.030	0.019	0.051	0.051	0.043	0.041	0.070	0.017
	$L2$	0.070	0.064	0.107	0.106	0.076	0.080	0.104	0.064
X	$L1$	0.062	0.088	-0.160	-0.002	-0.071	-0.443	-0.393	-0.234
	$L2$	0.059	0.092	-0.149	0.015	-0.063	-0.446	-0.388	-0.239

Electronic Properties

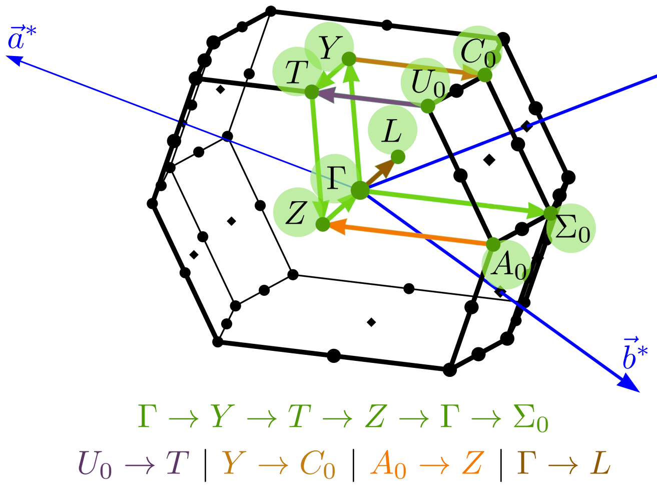

Label	Scaled coordinates
Γ	$(0, 0, 0)$
Y	$(1/2, 0, 1/2)$
T	$(1, 1/2, 1/2)$
Z	$(1/2, 1/2, 0)$
Σ_0	$(0, 0.3107, 0.3107)$
U_0	$(1, 0.6893, 0.6893)$
C_0	$(1/2, 0.2218, 0.7218)$
A_0	$(1/2, 0.7782, 0.2782)$
L	$(1/2, 1/2, 1/2)$

Figure S1: Left: Representation of the first Brillouin zone of the considered MOFs with the reciprocal axis indicated in blue and the high-symmetry points highlighted in green. The paths considered for plotting the band structures are reported at the bottom. Right: Scaled coordinates for the high-symmetry points marked on the right.

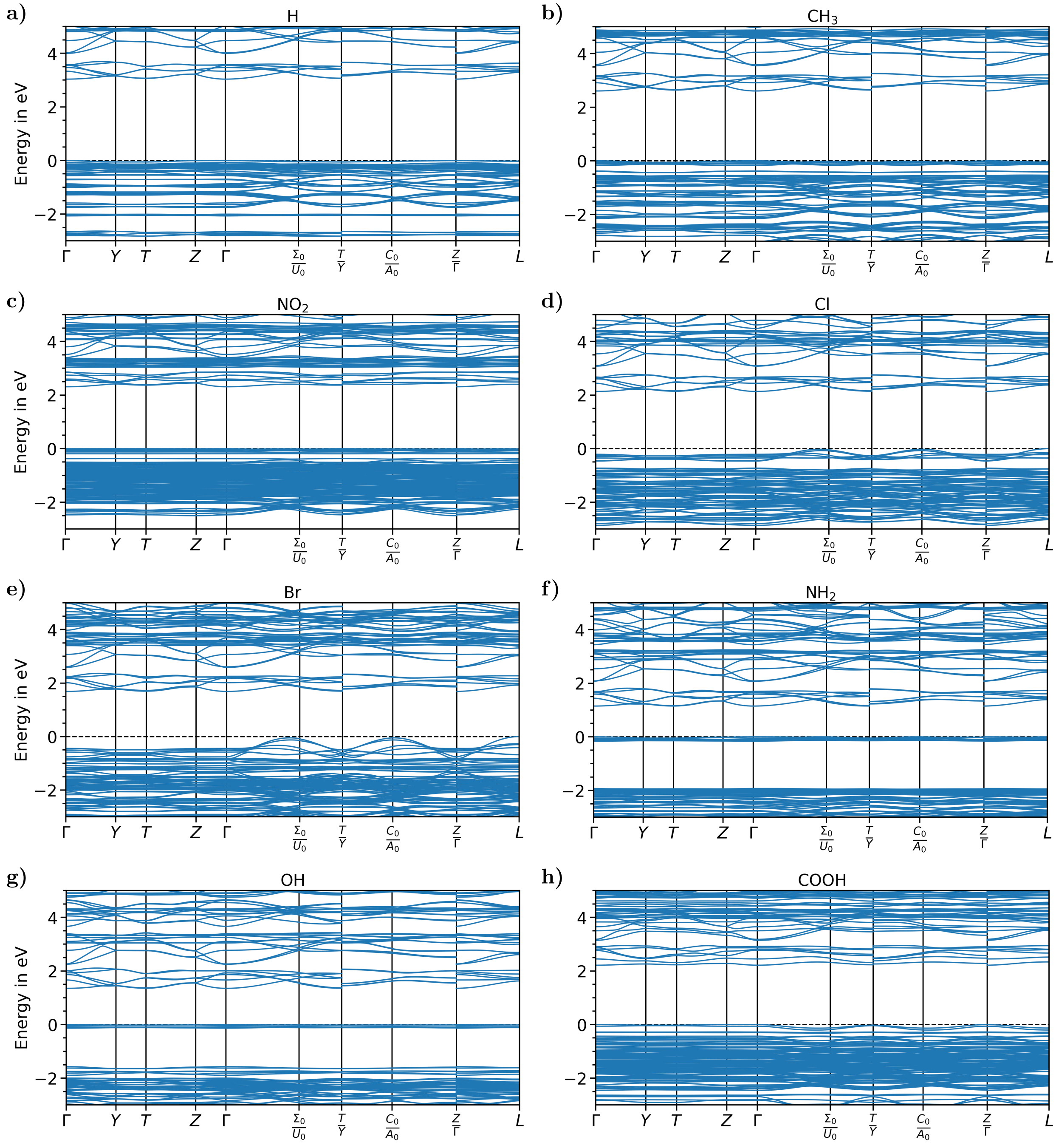

Figure S2: Band structures of the Sc-based MOFs with the indicated functionalizations.

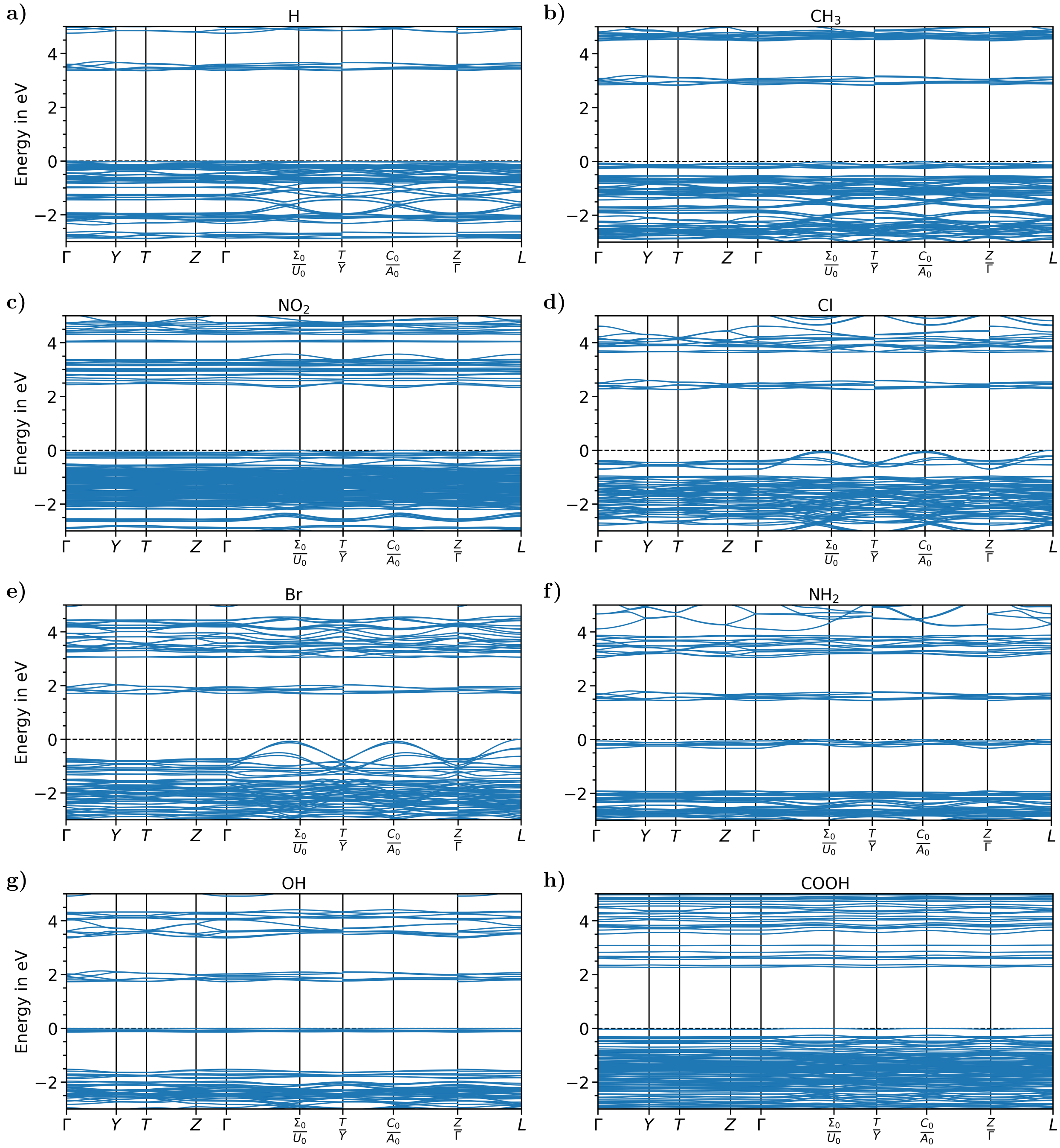

Figure S3: Band structures of the Al-based MOFs with the indicated functionalizations.

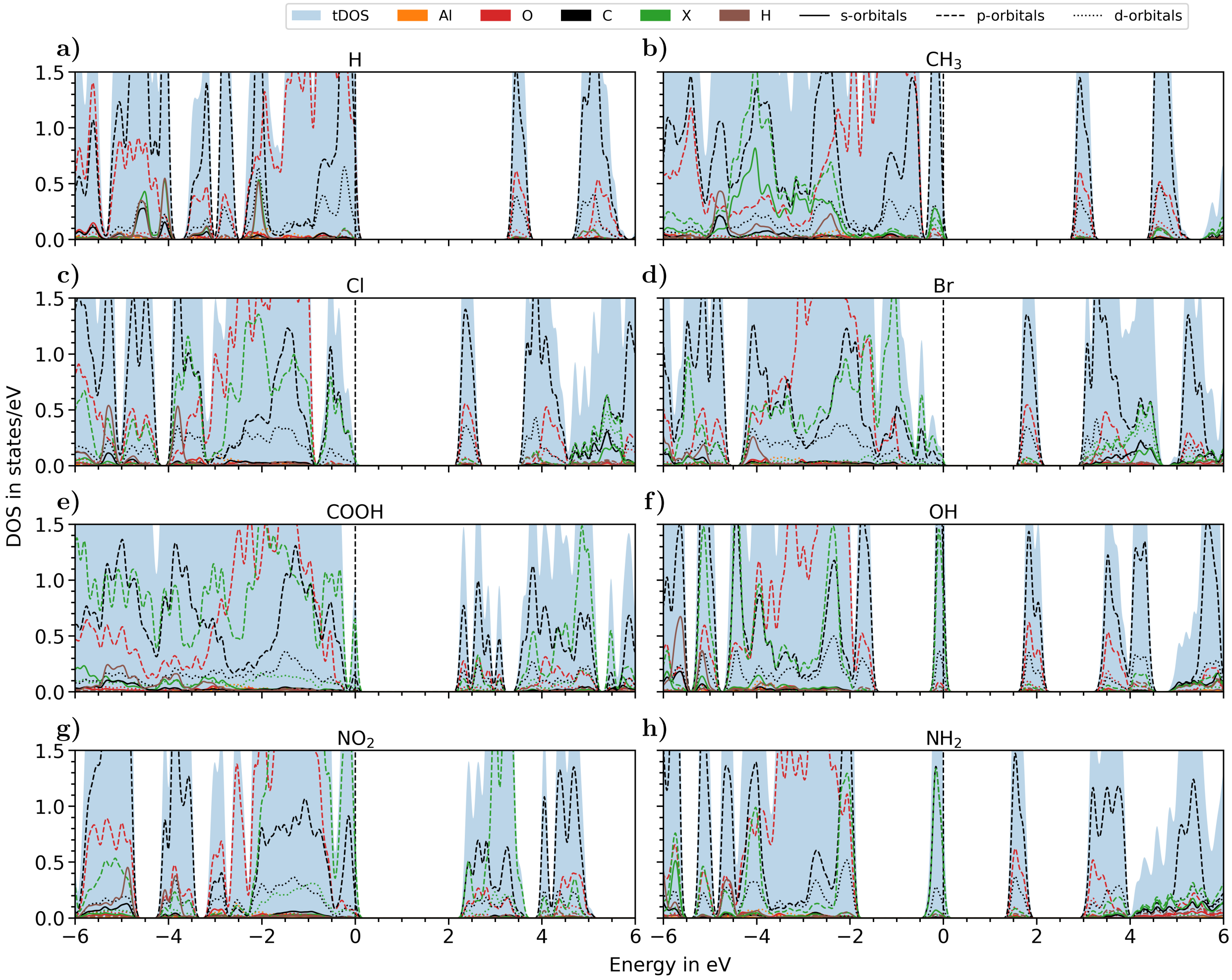

Figure S4: Atom-resolved pDOS of the Al-based MOFs with the indicated functionalizations. In the legend, M indicates the metal node, and X the functional group.

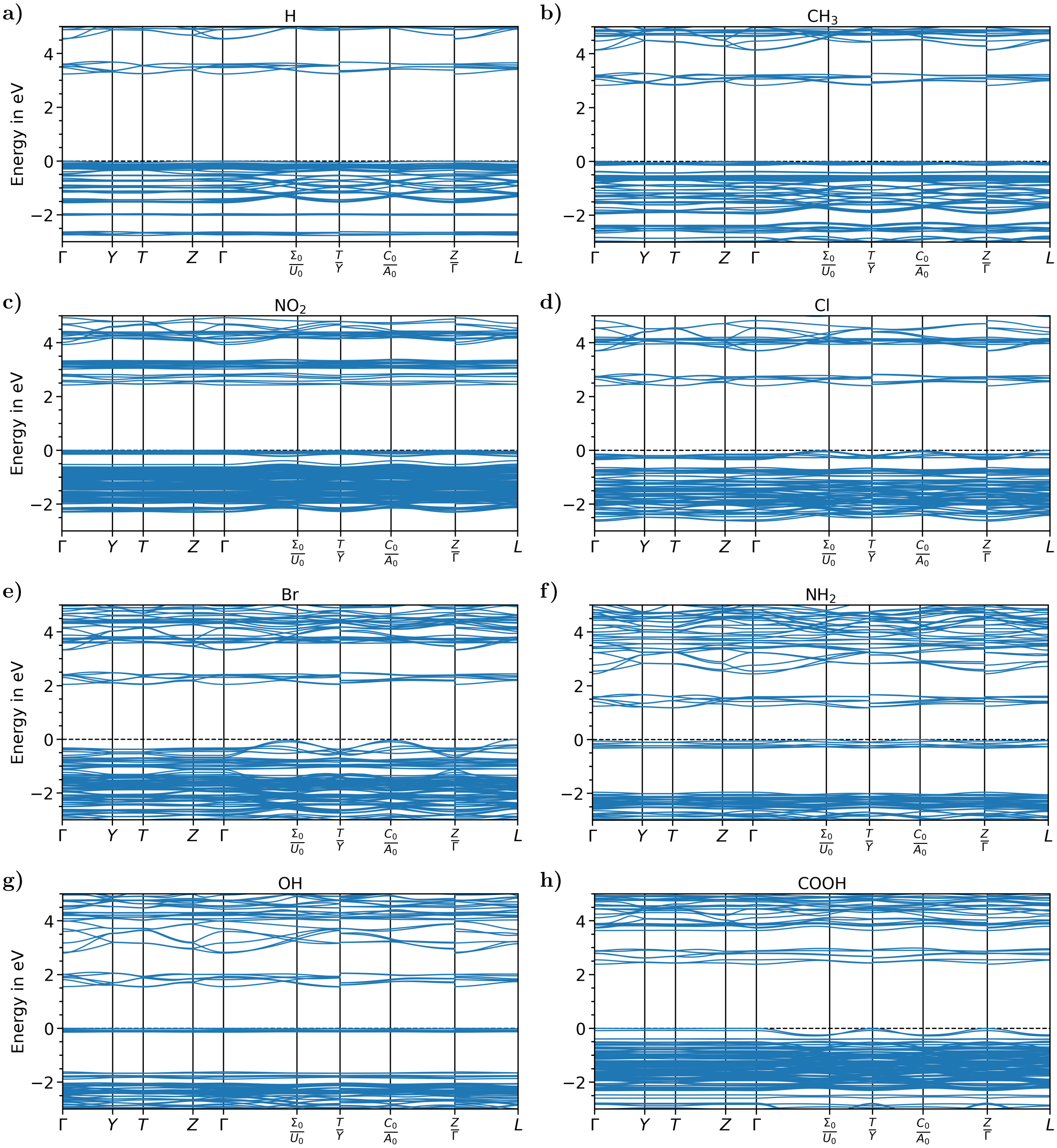

Figure S5: Band structures of the Y-based MOFs with the indicated functionalizations.

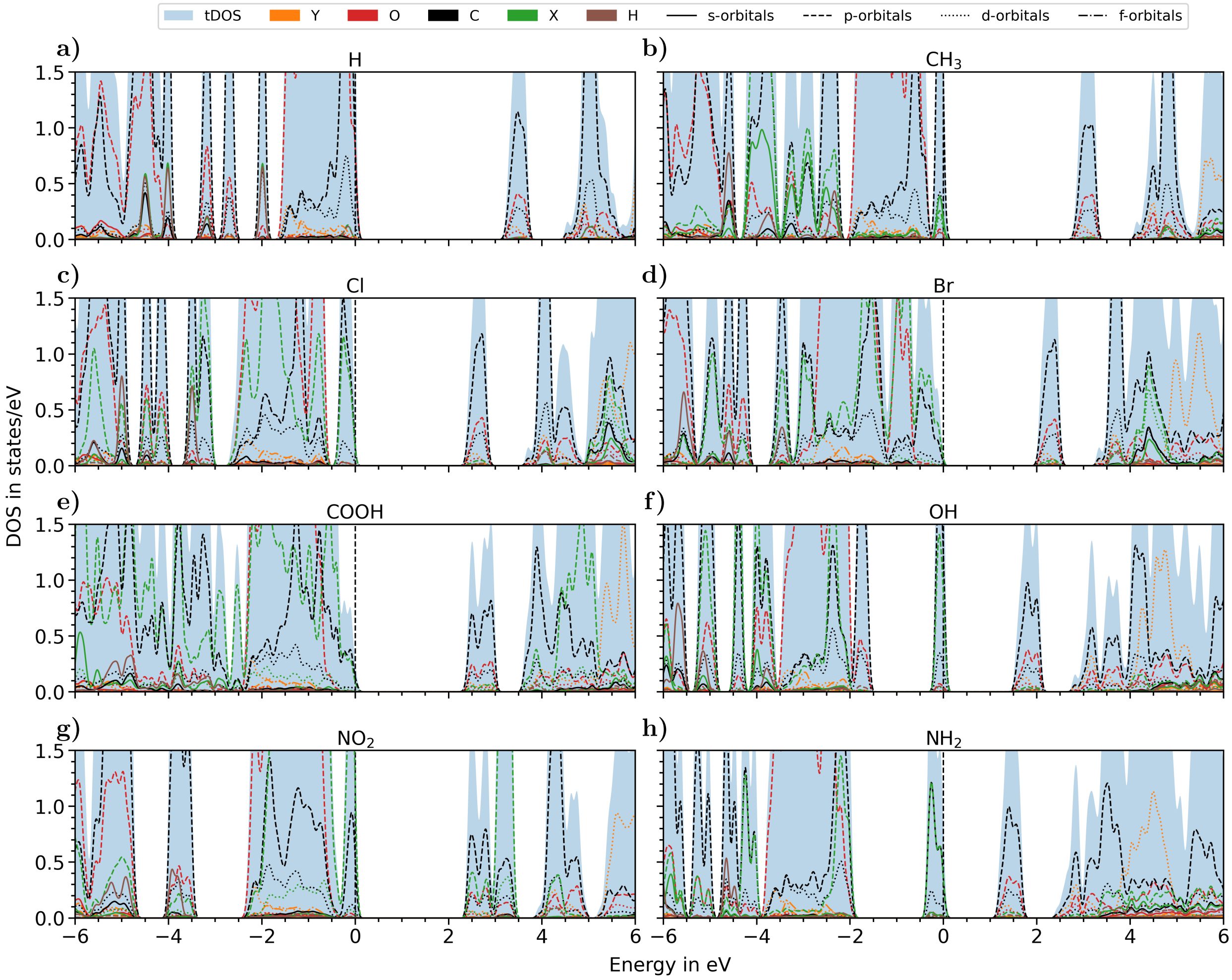

Figure S6: Atom-resolved pDOS of the Y-based MOFs with the indicated functionalizations. In the legend, M indicates the metal node, and X the functional group.
